# BaFe_12_O_19_-chitosan Schiff-base Ag (I) complexes embedded in carbon nanotube networks for high-performance electromagnetic materials

**DOI:** 10.1038/srep12544

**Published:** 2015-07-28

**Authors:** Jie Zhao, Yu Xie, Dongsheng Guan, Helin Hua, Rong Zhong, Yuancheng Qin, Jing Fang, Huilong Liu, Junhong Chen

**Affiliations:** 1 College of Environment and Chemical Engineering, Nanchang Hangkong University, Nanchang, 330063, PR China; 2 Department of Mechanical Engineering, University of Wisconsin-Milwaukee, Milwaukee, 53211, USA

## Abstract

The multiwalled carbon nanotubes/BaFe_12_O_19_-chitosan (MCNTs/BF-CS) Schiff base Ag (I) complex composites were synthesized successfully by a chemical bonding method. The morphology and structures of the composites were characterized with electron microscopy, Fourier transform infrared spectroscopy and X-ray diffraction techniques. Their conductive properties were measured using a four-probe conductivity tester at room temperature, and their magnetic properties were tested by a vibrating sample magnetometer. The results show that the BF-CS Schiff base Ag (I) complexes are embedded into MCNT networks. When the mass ratio of MCNTs and BF-CS Schiff base is 0.95:1, the conductivity, *M*_*s*_ (saturation magnetization), *M*_*r*_ (residual magnetization), and *H*_*c*_ (coercivity) of the BF-CS Schiff base composites reach 1.908 S cm^−1^, 28.20 emu g^−1^, 16.66 emu g^−1^ and 3604.79 Oe, respectively. Finally, a possible magnetic mechanism of the composites has also been proposed.

Nowadays magnetic materials have drawn extensive attention due to their potential applications in various fields such as catalysis^1^, optics^2^, separation^3^, enrichment^4^,^5^ and magnetic properties^6^,^7^,^8^,^9^. Among them, BaFe_12_O_19_ (BF) is an important magnet material because of its high Curie temperature, large saturation magnetization (*M*_*s*_), high magneto-crystalline anisotropy, good corrosion resistivity and excellent chemical stability^10^,^11^. It has been widely used in electromagnetic microwave absorbers^12^,^13^, magnetic fluids^14^, density perpendicular recording media^15^ and high frequency devices^16^,^17^. Some groups reported the combination of BF with electric materials to achieve novel composites with both magnetic and conductive properties^18^,^19^,^20^. However, the overall magnetic properties of these composites unfortunately decrease due to the lack of magnetic behaviors of the original electric materials according to the equation *M*_*s*_ = φm_s_^21^ (*M*_*s*_ is related to the volume fraction of whole particles (φ) and the saturation moment of a single particle (m_s_)). In addition, Schiff-base metal complexes with high *M*_*s*_ and conductivity are of great interest due to their lower density, easy processing, wider absorption bands as well as excellent microwave absorbing properties^22^,^23^,^24^,^25^,^26^,^27^. The Schiff-base metal complexes coated onto the surface of BF may slow down the deterioration of the overall magnetic properties of the final composites. Therefore, we proposed to replace these nonmagnetic electric materials with the Schiff-base metal complexes in the composites to make them show both magnetic and conductive behaviors.

It is also known that chitosan (CS) Schiff-base is a type of important organic-magnetic material due to its low-cost possessing, environmental friendliness and inherent chirality^28^,^29^,^30^,^31^. Its excellent electric and magnetic properties result from charge transfer along the -C=N- group and the magnetic moment formed by the electronic rotation of -C=N-, respectively^32^,^33^,^34^. Hence, CS Schiff-base has been chosen to adjust the overall magnetic properties of the final composites. Therefore, we attempted to graft the CS Schiff-base onto the surface of BF to obtain enhanced magnetic and conductive properties. However, the conductivity of BF-CS Schiff-base composites is still poorer than common semiconductor materials. Presently, carbon nanotubes (CNTs) are well known for their high aspect ratio, high Young’s modulus, chemical stability, nanometric dimensions and good electrical properties^35^. CNTs as dopant materials to improve the conductivity of electromagnetic composites have been reported. Wu’s group prepared CNTs/carbonyl iron complex absorbers and found their conductivity to be 13.54 S cm^−1^ when the ratio of CNTs is 6.6%^36^. Poly((α-methylstyrene)-co-butylmethacrylate) grafted multi-walled CNTs (MCNTs) with conductivity of 1.695 S cm^−1^ were synthesized by Liu *et al.*^37^. Wang’s group reported that the hybrid super-aligned carbon nanotube/carbon black conductive networks deliver excellent electrical conductivity and capacity in lithium ion batteries^38^. Therefore, we used MCNTs to build the conductive networks and absorb onto the surface of the BF-CS Schiff-base composites to prepare MCNTs/BF-CS Schiff base composite materials. In addition, Ag (I) metal ions coordinated with N atoms of the -CH=N- group can change the permeability and permittivity of Schiff base to gain improved electromagnetic properties^39^, so we further fabricate the MCNTs/BF-CS Schiff base Ag (I) complexes with a conductive network structure under mild conditions.

The as-prepared MCNTs/BF-CS Schiff base Ag (I) complexes exhibit excellent conductivity and relatively high M_s_ compared to the pure BF-CS Schiff base. The reason for the enhancement was studied, and the magnetic mechanism is proposed according to the effects of each component in the composites. Our work reveals the important role of conductive networks in the optimization of electromagnetic materials.

## Experimental

### Materials

BF was synthesized by the reported method^40^. CS (with a degree of deacetylation >90.0%) was purchased from Sinopharm Chemical Reagent Co. Ltd. MCNTs ( multiwalled, OD 20–30 nm, length 10–30 μm, purity >95%, ash <0.5 wt%, SSA > 200 m^2^ g^−1^, EC > 10^2^ S cm^−1^) were purchased from Beijing DK nano technology Co. Ltd. BF-CS Schiff base was prepared according to the method in the supporting information. Other chemicals were analytical grade.

### Purification of MCNTs

MCNTs were added into concentrated nitric acid and refluxed for 5 h at 90 ^o^C, and the precipitate was filtered and washed with 0.1 mol L^−1^ HCl and deionized water three times. Finally, the product was dried under vacuum at 50 ^o^C for 24 h.

### Synthesis of BF-CS Schiff base

2.0 g chitosan was dissolved into 50 mL diluted acetic acid (pH = 1). Then 2.0 g BF was added into the above solution with ultrasonic treatment for 0.5 h. After that, a 10 mol/L NaOH solution was slowly dropwise added into the above solution until the pH value of the system equaled 13. The mixture was heated to 60 ^o^C. Then, two drops of the 25% glutaraldehyde were added into the mixture stirring for 2 h. Finally, the precipitate was filtrated and washed with deionized water, ethanol, and acetone, respectively. The product was dried under vacuum at 50 ^o^C for 12 h. After that, 1.0 mL glyoxal was added into 80 mL absolute ethanol, stirring for 10 min. Then, 2.0 g of the above product was added into the above solution refluxing for 12 h at 75 ^o^C. After that, the precipitate was filtered and washed with ethanol 3 times. Finally, the precipitate was dried under vacuum at 30 ^o^C for 10 h.

### Preparation of MCNTs/BF-CS Schiff base Ag (I) complexes

1.0 g BF-CS Schiff base and 0.15 g purified MCNTs were added into 60 mL 10% NaOH and refluxed for 4 h at 40 ^o^C, and the precipitate was filtered and washed with deionized water until pH equaled 7. Then, the product was dried under vacuum at 50 ^o^C for 10 h. 0.05 g AgNO_3_ and the as-obtained product were added into 150 mL absolute ethanol and refluxed for 30 min at 85 ^o^C, and the precipitate was filtered and washed with absolute ethanol. The final product was dried under vacuum at 50 ^o^C for 12 h. We designated it as MCNTs/BF-CS Schiff base Ag (I) complexes (0.15:1) because the mass ratio of MCNTs and the BF-CS Schiff base Ag (I) complexes was 0.15:1. Two more samples, MCNTs/BF-CS Schiff base Ag (I) complexes (0:1; 0.55:1 and 0.95:1) were also synthesized with the same method.

### Characterization

Fourier transform infrared (FTIR) spectra were obtained using a Nicolet 5700 FTIR with the KBr method. X-ray diffraction (XRD) patterns of the samples were conducted by using a Philps-pw3040/60 diffractometer with Cu Kα radiation (λ = 0.15418 nm). The morphologies and microstructure of the samples were observed by a scanning electron microscope (SEM, Nova NanoSEM450) and a transmission electron microscope (TEM, JEOL JEM2010), respectively. The electrical conductivities were measured with a four-probe resistivity instrument (SDY-4) at room temperature using pressed pellets of sample powder with a thickness of about 1 mm and a diameter of 1 cm. A Lakeshore 7404 vibrating sample magnetometer was used to measure the magnetization in applied magnetic fields within the range of −10 to +10 kOe at room temperature.

## Results and Discussion

### Synthesis route of MCNTs/BF-CS Schiff base Ag (I) complexes

**Figure 1:**
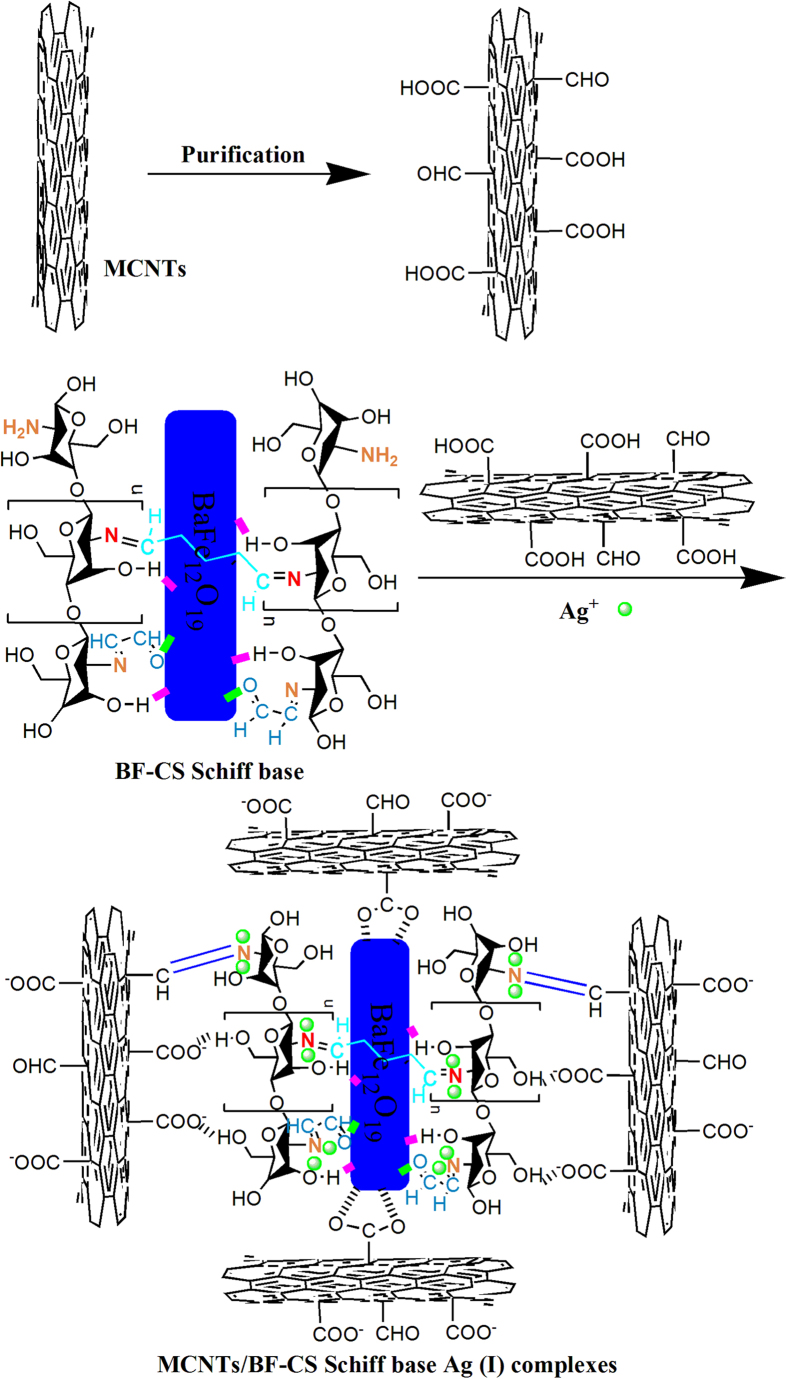
The synthesis route of the MCNTs/BF-CS Schiff base Ag (I) complexes.

Figure 1 illustrates the synthesis route of MCNTs/BF-CS Schiff base Ag (I) complexes. The MCNTs/BF-CS Schiff base Ag (I) complexes are prepared through a chemically bonded method using purified MCNTs with a large number of -COO- surface groups. MCNTs are adsorbed onto the surface of BF-CS Schiff base Ag (I) complexes by hydrogen bonding interactions between MCNTs and BF-CS Schiff base, so BF-CS Schiff base Ag (I) complexes are successfully embedded into the MCNT network. The network can certainly provide numerous electronic transfer paths to enhance the conductive behaviors of the composite. Moreover, the combination of inorganic magnet BF with organic magnet CS Schiff-base may yield an outstanding magnet with a balance of permittivity and permeability. Meanwhile, Ag (I) metal ions coordinated with N atoms of the -CH=N- groups can adjust the permeability and permittivity of the Schiff base to gain optimal electromagnetic properties^39^. Hence, the MCNTs/BF-CS Schiff base Ag (I) complexes with excellent magnetic and conductive performance are expected.

### XRD analysis

**Figure 2:**
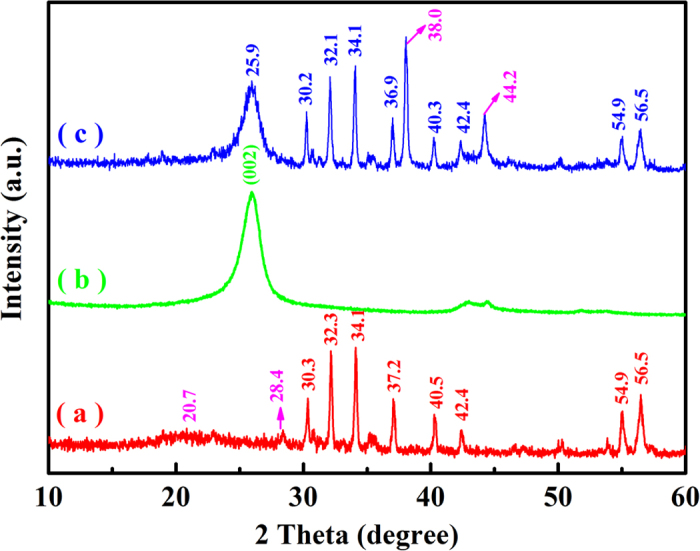
XRD patterns of (**a**) BF-CS Schiff base, (**b**) MCNTs and (**c**) MCNTs/BF-CS Schiff base Ag (I) complexes (0.95:1).

The XRD analysis of the MCNTs/BF-CS Schiff base Ag (I) complexes is shown in Fig. 2. The characteristic diffraction peaks of the BF-CS Schiff base composites are at 2θ = 20.7°, 28.4°, 30.3°, 32.3°, 34.1°, 37.2°, 40.5°, 42.4°, 54.9° and 56.5°. BF has characteristic diffraction peaks at 2θ = 30.3° (110), 32.3° (107), 34.1° (114), 37.2° (203), 40.5° (205), 54.9° (217) and 56.5° (2011) (PDF#00–043–002, hexagonal, P63/mmc). Peaks at 2θ of 20.7° and 28.4° can be indexed to CS and CS Schiff base, suggesting that the BF-CS Schiff base composites have been synthesized successfully^19^. In Fig. 2b, the peak at 2θ = 25.9° can be ascribed to the (002) reflection of MCNTs^41^. As shown in Fig. 2c, it is clearly seen that MCNTs have a peak at 25.8° (002), and the BF-CS Schiff base composites have peaks at 30.2°, 32.1°, 34.1°, 36.9°, 40.3°, 42.4°, 54.9° and 56.5°. Compared to Fig. 2a,b, new peaks at 2θ = 38.0° and 44.2° are found in Fig. 2c, which may be due to the complexes formed by Ag (I) ions and electronic pairs of N atoms in the -C=N- groups of CS Schiff bases^42^. The results indicate that the MCNTs/BF-CS Schiff base Ag (I) complexes have been obtained and embedded in the networks of MCNTs.

### Chemical bonding between components

**Figure 3:**
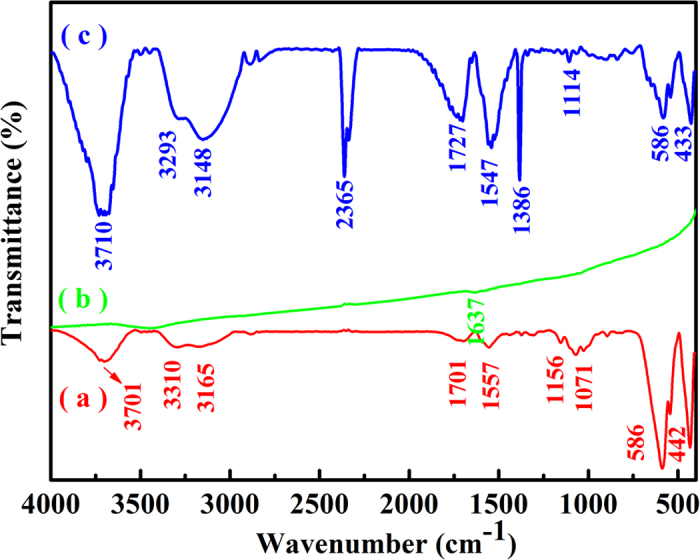
FTIR spectra of (**a**) BF-CS Schiff base, (**b**) MCNTs and (**c**) MCNTs/BF-CS Schiff base Ag (I) complexes (0.95:1).

Figure 3 gives FTIR spectra of (a) BF-CS Schiff base, (b) MCNTs and (c) MCNTs/BF-CS Schiff base Ag (I) complexes. Figure 3a shows the characteristic absorption peaks of BF-CS Schiff base composites at 3701, 3310, 3165, 1701, 1557, 1156, 1071, 586 and 442 cm^−1^. Peaks at 3701, 3310, 3165 and 1701 cm^−1^ can be assigned to O-H, hydrogen bonds, N-H and C=C-C=O stretching vibration, respectively^43^. The peak at 1557 cm^−1^ can be assigned to C=N stretching vibration^44^, indicating that the CS Schiff base has been formed. Peaks at 1156 and 1071 cm^−1^ are attributed to the C-O and C-C stretching vibration, respectively^45^,^46^. The peaks at 586 and 442 cm^−1^ can be assigned to Fe(Ba)-O stretching vibration^40^. The above results reveal that the BF-CS Schiff base composites have been formed through hydrogen bonds between them. The characteristic peak of MCNTs (Fig. 3b) is weak at 1637 cm^−1^ due to the C=C symmetrical stretching vibration^20^. In Fig. 3c, two new peaks at 2365 and 1386 cm^−1^ have been identified for the MCNTs/BF-CS Schiff base Ag (I) complexes, and the peak of C=N stretching vibration shifts from 1557 to 1547 cm^−1^ compared to that in Fig. 3a. Peaks at 2365 and 1386 cm^−1^ are assigned to -C=C=C- and C-H out-of-plane stretching vibration, which shows that the BF-CS Schiff base is embedded in the networks of MCNTs. The red shift of the C=N stretching vibration peak is derived from the complexation of Ag (I) ions with N atoms, suggesting that the MCNTs/BF-CS Schiff base Ag (I) complexes have been synthesized. Peaks at 3710, 3293, 3148, 1727 and 1114 cm^−1^ can be assigned to O-H, hydrogen bonds, N-H, C=C-C=O and C-C stretching vibration, respectively. Fe(Ba)-O stretching vibrations are at 586 and 433 cm^−1^.

### Microstructures of the composites

**Figure 4:**
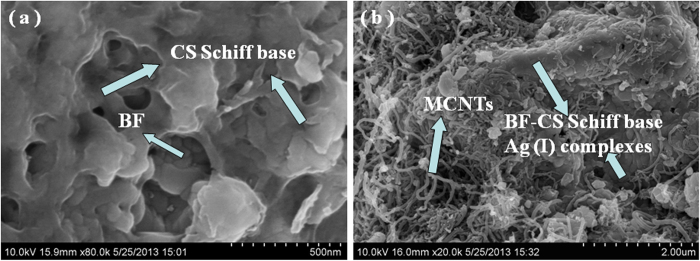
SEM images of (**a**) BF-CS Schiff base and (**b**) MCNTs/BF-CS Schiff base Ag (I) complexes (0.95:1).

SEM and TEM have been employed to observe the microstructure of composites. As shown in Fig. 4a, the SEM image directly shows that the BF-CS Schiff-base composite (without MCNTs) is assembled by aggregation of BF and CS particles. It seems that the BF particles are almost covered by the CS Schiff-base. In Fig. 4b, the BF-CS Schiff base Ag (I) complexes as the component of the MCNTs/BF-CS Schiff base Ag (I) complexes have been produced. The BF-CS Schiff base Ag (I) complexes are inserted into the networks of MCNTs. The component of the BF-CS Schiff base and the MCNTs/BF-CS Schiff base Ag (I) complexes have been marked out in Fig. 4a,4b, respectively. The magnetic properties of the MCNTs/BF-CS Schiff base Ag (I) complexes are attributed to the combined effect of MCNTs and the BF-CS Schiff base Ag (I) complexes.

**Figure 5:**
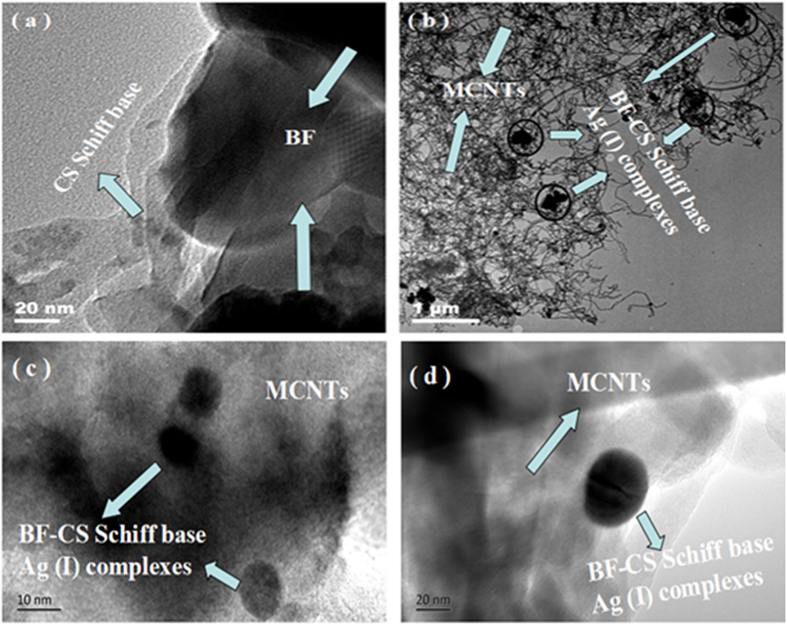
TEM images of the BF-CS Schiff base (**a**) and The MCNTs/BF-CS Schiff base Ag (I) complexes (0.95:1) (**b–d**).

TEM images of the BF-CS Schiff base and the MCNTs/BF-CS Schiff base Ag (I) complexes (0.95:1) are shown in Fig. 5. The irregular CS Schiff base seen in Fig. 5a indicates that the BF-CS Schiff base composites have been produced. The black components are BF, and CS Schiff base locates on the surface of BF. As shown in Fig. 5b–d, the BF-CS Schiff base Ag (I) complexes, as an inorganic-organic hybrid magnetic material, are embedded uniformly into the MCNT network. The network provides paths for electron transfer, which is helpful for improving the conductivity of the MCNTs/BF-CS Schiff base Ag (I) complexes. Therefore, we can conclude that the MCNTs/BF-CS Schiff base Ag (I) complexes with network structures have been prepared.

**Figure 6:**
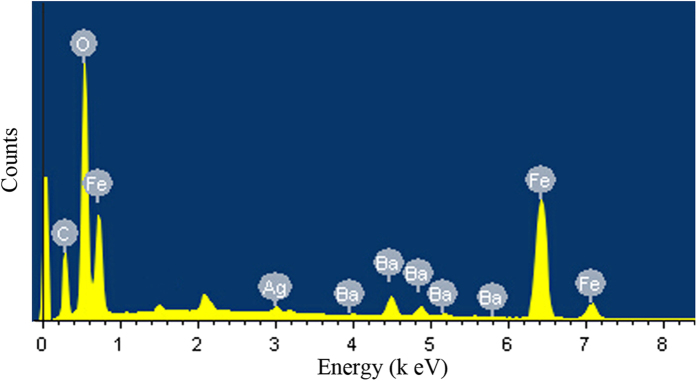
EDS spectrum of MCNTs/BF-CS Schiff base Ag (I) complexes (0.15:1).

The elemental composition of MCNTs/BF-CS Schiff base Ag (I) complexes has been analyzed by EDS. Figure 6 gives the typical EDS spectrum of the MCNTs/BF-CS Schiff base Ag (I) complexes (0.15:1). Elements including C, O, Fe, Ag and Ba are observed. The Ba peaks derive from the BF, and Ag comes from the Schiff-base Ag (I) complexes. A quantitative analysis reveals that the mass ratio of C, O, Fe, Ba and Ag elements is around 30.45%, 45.50%, 21.67%, 1.70% and 0.33%, respectively.

### Conductivity properties

**Figure 7:**
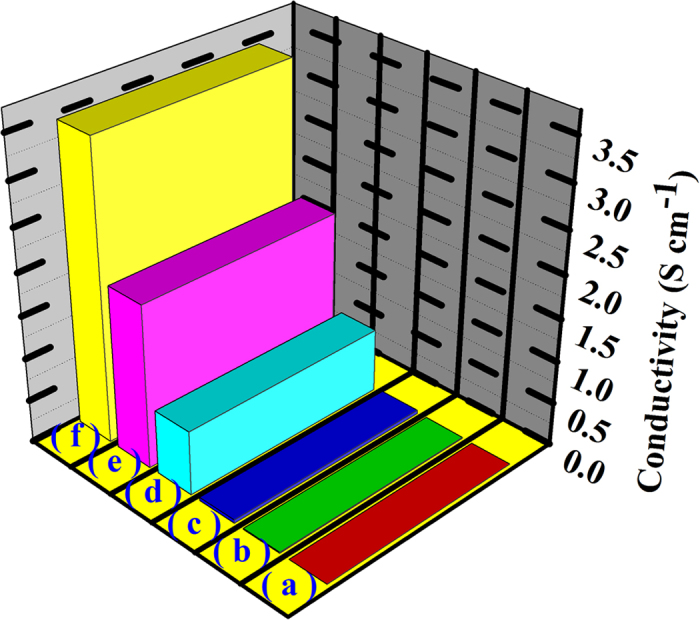
The electrical conductivity of (**a**) BF-CS Schiff base, (**b**) BF-CS Schiff base Ag (I) complexes, (**c**) MCNTs/BF-CS Schiff base Ag (I) complexes (0.15:1), (**d**) MCNTs/BF-CS Schiff base Ag (I) complexes (0.55:1), (e) MCNTs/BF-CS Schiff base Ag (I) complexes (0.95:1) and (f) MCNTs.

**Figure 8:**
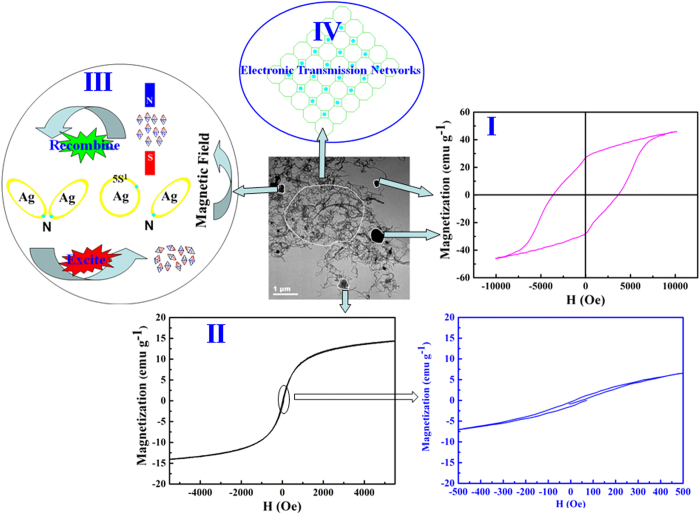
The proposed magnetic and conductive mechanism of MCNTs/BF-CS Schiff base Ag (I) complexes.

Figure 7 shows the electrical conductivity measurements of (a) BF-CS Schiff base, (b) BF-CS Schiff base Ag (I) complexes, (c) MCNTs/BF-CS Schiff base Ag (I) complexes (0.15:1), (d) MCNTs/BF-CS Schiff base Ag (I) complexes (0.55:1), (e) MCNTs/BF-CS Schiff base Ag (I) complexes (0.95:1) and (f) MCNTs. The conductivity values are summarized in Table 1. The conductivity of the BF-CS Schiff base is only 0.002 S cm^−1^, because the BF is an insulator and only CS Schiff base contributes to the total conductivity. From Fig. 7, we can see that the conductivity of MCNTs/BF-CS Schiff base Ag (I) complexes increases with the contents of MCNTs. When the mass ratio of MCNTs and BF-CS Schiff base is 0.15:1, the conductivity of MCNTs/BF-CS Schiff base Ag (I) complexes reaches 0.037 S cm^−1^. When the ratio is 0.95:1, their conductivity goes up to 1.908 S cm^−1^. Three possible reasons account for the conductivity changes of MCNTs/BF-CS Schiff base Ag (I) complexes. First, the CS Schiff base contributes to the total conductivity. Second, BF-CS Schiff base Ag (I) complexes enhance the conductivity of composites due to doping effects of Ag (I) ions. Third, the MCNT network in the MCNTs/BF-CS Schiff base Ag (I) complexes (Fig. 8IV) drastically improves their conductivity. The more the MCNTs are there, the bigger the improvement is.

### Magnetic property measurements

**Figure 9:**
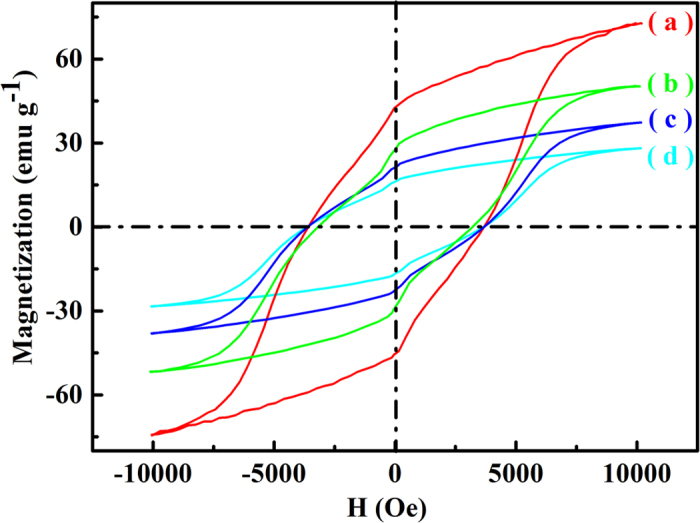
Magnetization hysteretic loops for (**a**) BF-CS Schiff base, (**b**) MCNTs/BF-CS Schiff base Ag (I) complexes (0.15:1), (**c**) MCNTs/BF-CS Schiff base Ag (I) complexes (0.55:1) and (**d**) MCNTs/BF-CS Schiff base Ag (I) complexes (0.95:1).

The magnetic properties of all samples were tested at room temperature and all of them exhibit clear hysteretic behaviors. Their hysteretic loops are shown in Fig. 9, and the magnetic parameters are summarized in Table 2. The *M*_*s*_, *M*_*r*_, and *H*_*c*_ of the BF-CS Schiff base composites reach 75.07 emu g^−1^, 45.20 emu g^−1^ and 3617.32 Oe, respectively. This is mainly due to the BF magnets that induce the magnetic properties of the whole composites. However, the *M*_*s*_ and *M*_*r*_ of MCNTs/BF-CS Schiff base Ag (I) complexes decrease with the increasing amount of MCNTs. When the mass ratio of MCNTs and BF-CS Schiff base is 0.95:1, the *M*_*s*_, *M*_*r*_, and *H*_*c*_ of the BF-CS Schiff base composites reach 28.20 emu g^−1^, 16.66 emu g^−1^ and 3604.79 Oe, respectively. According to previous reports^21^, it is understood that a non-magnetic CS coating layer and soft magnetic MCNTs impede the total magnetization and cause a decrease in the saturation magnetization. In addition, the *H*_*c*_ of MCNTs/BF-CS Schiff base Ag (I) complexes initially drops and then goes up owing to the influence of MCNTs. The detailed analysis of the magnetic mechanism of MCNTs/BF-CS Schiff base Ag (I) complexes will be discussed as follows.

The proposed magnetic mechanism of the MCNTs/BF-CS Schiff base Ag (I) complexes is shown in Fig. 8(I-III). There are at least three factors related to the overall magnetic performance. First, BF as an inorganic magnetic component in the composites plays an important role for the total magnetic properties. Figure 8I shows the BF hysteretic behaviors with *M*_*s*_, *M*_*r*_, and *H*_*c*_ of 46.04 emu g^−1^, 28.62 emu g^−1^ and 3610.15 Oe, respectively. In addition, MCNTs with *M*_*s*_ about 14.02 emu g^−1^ possess soft magnetic behaviors (Fig. 8II) due to the quantum size and microscopic aggregates. Moreover, Schiff-base metal complexes also contribute to the magnetic properties. In Fig. 8III, when the Ag (I) complexes are excited, the electron of N atoms jumps into the 5s orbit of Ag to generate magnetic moments. When a magnetic field is applied, the Schiff base Ag (I) complexes perform obviously magnetic behaviors. This is due to the fact that electrons are the primary stakeholder for magnetic materials, and the total magnetic moments of atoms are the sum of electronic orbital and spin magnetic moments. Therefore, the magnetic mechanism tends to be complicated and diversified for the MCNTs/BF-CS Schiff base Ag (I) complexes. In summary, the magnetic properties of the MCNTs/BF-CS Schiff base Ag (I) complexes are a result of the relationships between BF, the CS Schiff-base Ag(I) complex and MCNTs.

## Conclusions

The MCNTs/BF-CS Schiff base Ag (I) complexes have been synthesized successfully. FTIR and XRD patterns reveal the combination of BF-CS Schiff base Ag (I) complexes and MCNTs. The microstructure of the composites can be seen clearly by SEM and TEM. BF-CS Schiff base Ag (I) complexes are embedded into the networks of MCNTs through hydrogen bonds and conjugate interactions. When the mass ratio of MCNTs and BF-CS Schiff base is 0.95:1, the conductivity, *M*_*s*_, *M*_*r*_, and *H*_*c*_ of the BF-CS Schiff base composites reach 1.908 S cm^−1^, 28.20 emu g^−1^, 16.66 emu g^−1^ and 3604.79 Oe, respectively. The MCNTs/BF-CS Schiff base Ag (I) complexes with relativity high *M*_*s*_, *M*_*r*_, *H*_*c*_ and excellent conductivity may attract increasing interest in various scientific and industrial fields. This study also provides a feasible way to readily optimize electromagnetic materials through inorganic-organic hybrid structures for magnetic applications.

## Additional Information

**How to cite this article**: Zhao, J. *et al.* BaFe_12_O_19_-chitosan Schiff-base Ag (I) complexes embedded in carbon nanotube networks for high-performance electromagnetic material. *Sci. Rep.* **5**, 12544; doi: 10.1038/srep12544 (2015).

## Figures and Tables

**Table 1:** The electrical conductivity of BF-CS Schiff base, BF-CS Schiff base Ag (I) complexes, MCNTs/BF-CS Schiff base Ag (I) complexes and MCNTs.

**Sample**	**Conductivity (S cm^−1^)**
BF-CS Schiff base	0.002
BF-CS Schiff base Ag (I) complexes	0.009
MCNTs/BF-CS Schiff base Ag (I) complexes (0.15:1)	0.037
MCNTs/BF-CS Schiff base Ag (I) complexes (0.55:1)	0.750
MCNTs/BF-CS Schiff base Ag (I) complexes (0.95:1)	1.908
MCNTs	3.472

**Table 2:** Magnetic parameters of BF-CS Schiff base and MCNTs/BF-CS Schiff base Ag (I) complexes.

**Sample**	***M*_*s*_ (emu g^-1^)**	***M*_*r*_ (emu g^-1^)**	***H*_*c*_ (Oe)**
BF-CS Schiff base	75.07	45.20	3617.32
MCNTs/BF-CS Schiff base Ag (I) complexes (0.15:1)	50.19	28.55	3218.27
MCNTs/BF-CS Schiff base Ag (I) complexes (0.55:1)	37.42	21.76	3502.79
MCNTs/BF-CS Schiff base Ag (I) complexes (0.95:1)	28.20	16.66	3604.79
